# Resveratrol-βcd inhibited premature ovarian insufficiency progression by regulating granulosa cell autophagy

**DOI:** 10.1186/s13048-024-01344-0

**Published:** 2024-01-15

**Authors:** Bingbing Hu, Xiushuang Zheng, Wei Zhang

**Affiliations:** 1 The Reproductive Medicine Center, The Seventh Affiliated Hospital, Sun Yat-Sen University, Shenzhen, China; 2 Department of Reproductive Medicine, The First Affiliated Hospital of Harbin Medical University, Harbin, China; 3 Emergency and Disaster Medical Center, The Seventh Affiliated Hospital, Sun Yat-Sen University, Shenzhen, China

**Keywords:** Resveratrol, Premature ovarian insufficiency (POI), Granulosa cells (GCs), Macrophage, IL-6

## Abstract

**Background:**

The ovarian environment of premature ovarian insufficiency (POI) patients exhibits immune dysregulation, which leads to excessive secretion of numerous proinflammatory cytokines that affect ovarian function. An abnormal level of macrophage polarization directly or indirectly inhibits the differentiation of ovarian granulosa cells and steroid hormone production, ultimately leading to POI. Resveratrol, as a health supplement, has been widely recognized for its safety. There is a substantial amount of evidence indicating that resveratrol and its analogs possess significant immune-regulatory functions. It has also been reported that resveratrol can effectively inhibit the progression of POI. However, the underlying immunological and molecular mechanisms through which resveratrol inhibits the progression of POI are still unclear.

**Results:**

Our preliminary reports have shown that resveratrol-βcd, the beta-cyclodextrin complex of resveratrol, significantly enhances the stability of resveratrol. Resveratrol-βcd could regulate the dysfunctional immune status of macrophages and T cells in the tumor microenvironment. In this study, we treated busulfan and cyclophosphamide (B/C)-treated mice, which were used as a POI model, with resveratrol-βcd. After resveratrol-βcd treatment, the levels of IL-6 in the ovaries were significantly increased, and the progression of POI was suppressed. IL-6 activated granulosa cells (GCs) through soluble IL-6R (sIL-6R), promoting autophagy in GCs. Resveratrol-βcd and IL-6 had a synergistic effect on enhancing autophagy in GCs and promoting E2 secretion.

**Conclusions:**

We partially elucidated the immune mechanism by which resveratrol inhibits the progression of POI and the autophagy-regulating function of GCs. This provides a theoretical basis for using resveratrol to prevent POI in future studies and clinical guidance.

**Supplementary Information:**

The online version contains supplementary material available at 10.1186/s13048-024-01344-0.

## Background

The development of ovaries is closely related to their normal immune environment. An abnormal immune environment can lead to ovarian dysfunction, such as premature ovarian insufficiency or polycystic ovarian syndrome (PCOS). POI refers to the continuous decline and dysfunction of ovarian function in women before the age of 40 [1, 2]. A recent report showed that the global overall prevalence of POI among women was 3.5% [3].

POI is a highly heterogeneous disease, and in most cases, the molecular pathophysiology of POI remains unexplained. Currently, POI is believed to be caused by genetic or other unknown factors, either single-factor or syndromic [4]. Granulosa cell (GCs) surround the oocytes and play a key role in follicle development and fate determination. In addition to providing essential nutrients and growth factors for follicular development, GCs also secrete steroid hormones required for folliculogenesis [5]. Dysfunction of GCs can lead to follicle arrest and apoptosis, ultimately resulting in POI [6, 7]. The cellular functions of GCs are strongly positively correlated with the maintenance of normal ovarian function [8].

Resveratrol, belonging to the stilbene subclass, is derived from phenylalanine and contains an aromatic ring with an active hydroxyl group [9]. It is a natural compound with free radical scavenging activity. In the female reproductive system, resveratrol can enhance the quality of oocytes through its antioxidant and anti-apoptotic functions [10]. Resveratrol can maintain the normal physiological function of ovarian granulosa cells [11]. Resveratrol also exerts its antagonistic effect on POI by modulating the immune response [12]. However, the specific cellular functions of ovarian granulosa cells affected by resveratrol remain unclear.

An increasing amount of evidence indicates that autophagy is closely related to the development of GCs. Autophagy is a major biological process involved in the degradation and recycling of cellular components and damaged organelles [13–15]. Autophagy also participates in regulating the apoptosis of GCs to accelerate follicular atresia, while insufficient granulosa-lutein cell autophagy leads to decreased progesterone synthesis and preterm birth in mice [15, 17]. GCs from patients with primary ovarian insufficiency (POI) show significant inhibition of autophagy levels, decreased expression of autophagy-related genes, a decrease in the ratio of LC3-II to LC3-I and an increase in p62 protein levels [16]. Autophagic flux blockade caused by Epg5 deficiency leads to decreased expression of steroidogenic genes, thereby interfering with GC differentiation [17].

In this study, we improved the stability and bioavailability of resveratrol by constructing resveratrol-βcd, which made it easier for the body to absorb resveratrol and achieve a higher C_max_ at a lower concentration. In a mouse model of POI, resveratrol-βcd restored the proportion and function of macrophages in the ovarian environment, inhibited the progression of POI, and maintained normal ovarian function. After resveratrol-βcd treatment, macrophage-derived IL-6 and resveratrol directly acted on GCs, promoting the autophagy level of GCs and enhancing their estrogen secretion function. Resveratrol-βcd inhibits the progression of POI by exerting combined effects on immune cells and GCs.

## Results

### Resveratrol-βcd can improve the stability of resveratrol

**Fig. 1:**
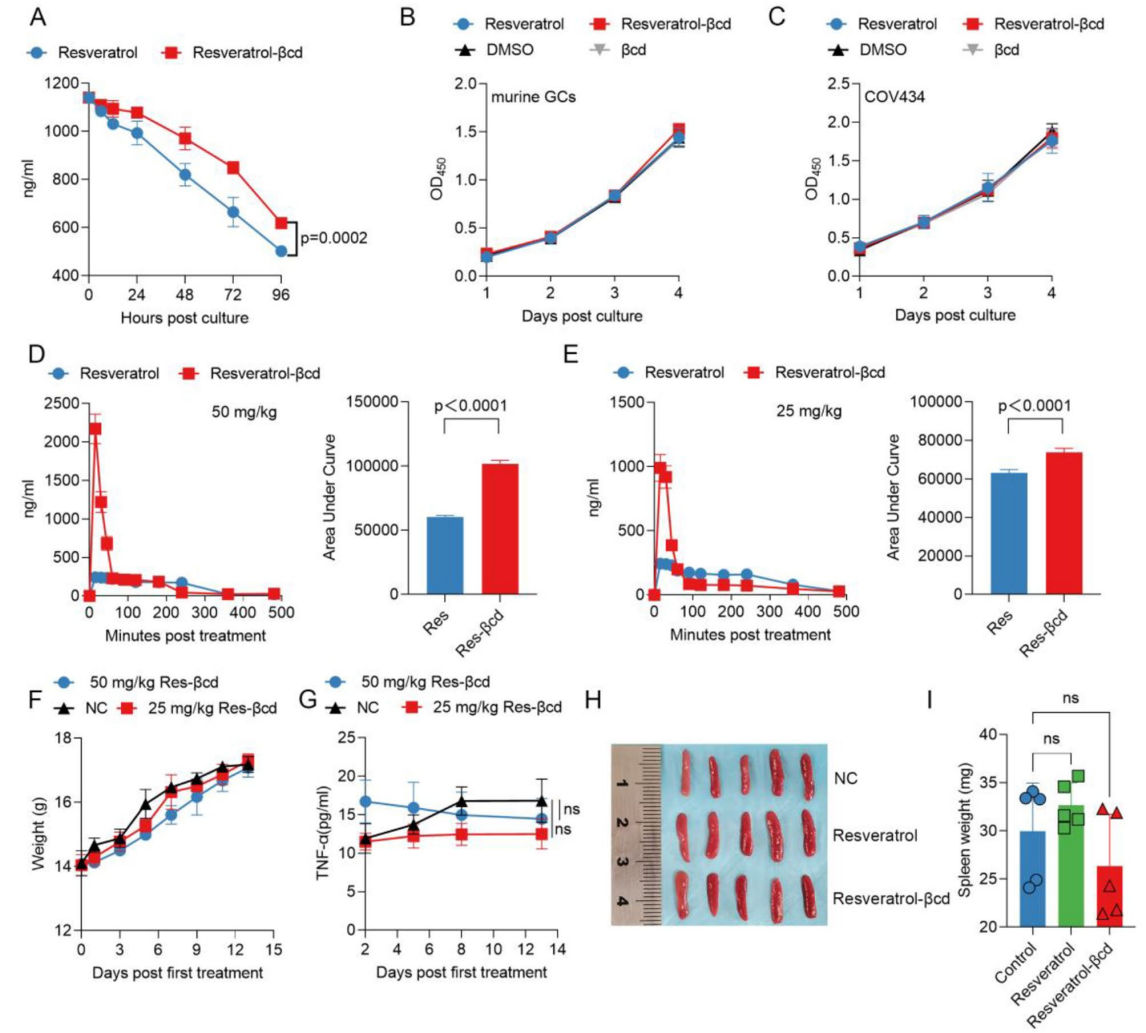
Analysis of the effects of resveratrol-βcd on the proliferation of ovarian granulosa cells and the immune response. **(A)** 6 ml DMEM culture medium was added to a 25 cm^2^ cell culture flask. For different groups, 5 µM (1141.2 ng/ml) resveratrol-βcd or resveratrol was added. 200 µl of supernatant at 6, 12, 24, 48, 72, and 96 h after incubation was collected, and the resveratrol concentration in the culture medium was detected using ELISA. *n* = 3; **(B)** Primary mouse granulosa cells or **(C)** COV434 cells were cultured with 5 µM resveratrol-βcd or resveratrol. The culture medium was replaced every 24 h to maintain the drug concentration. On days 1, 2, 3, and 4 after drug treatment, a CCK-8 assay kit was used to detect cell proliferation. *n* = 3; the statistical results are not significant among groups. **(D)** Resveratrol-βcd or resveratrol was administered by gavage at a dose of 50 mg/kg or **(E)** 25 mg/kg, and the levels of resveratrol in the blood were measured at corresponding time points after gavage. The statistical results of the AUC are on the right side of each line graph, *n* = 5; **(F)** Resveratrol-βcd or resveratrol was administered by gavage at a dose of 25 mg/kg or 50 mg/kg every other day, and the changes in body weight of the mice during the treatment period are shown, *n* = 5; the statistical results are not significant among groups. **(G)** The changes of TNFα in the serum during the treatment period. *n* = 5; **(H)** Typical images of mouse spleens on day 13 after drug administration and **(I)** their weights, *n* = 5. Each group used 5 mice for experiment, in vivo experiments in this study were independently repeated three times. Statistical analysis was performed using two-way ANOVA (**A,B,C,F,G**), and unpaired two-tailed Student’s t test (**D,E**). one-way ANOVA (**I**), comparison was made between control vs. resveratrol-βcd, control vs. resveratrol; ns, not significant

According to previous studies, we found that the resveratrol-βcd complex can enhance the solubility of trans-resveratrol [18]. However, it remains unclear whether the resveratrol-βcd complex can enhance the stability of resveratrol. We evaluated the detection of resveratrol-βcd using the resveratrol ELISA kit. The results showed that the resveratrol ELISA kit provided accurate measurements of resveratrol-βcd concentrations (Supplementary Fig. 1). We used the ELISA method to detect the decrease in resveratrol in the culture medium. Within a 48-hour period, the resveratrol content in the resveratrol-βcd group remained at 84.3%, which was a 9.5% improvement (*p* = 0.0002) compared to the resveratrol group’s 74.8% (Fig. 1A). Additionally, we evaluated the impact of resveratrol on cell proliferation in vitro. The results showed that at a concentration of 5 µM, resveratrol had almost no effect on the proliferation of primary ovarian granulosa cells and COV434 ovarian granulosa cells (Fig. 1B, C).

The pharmacokinetic parameters and safety of resveratrol in vivo was also evaluated. We performed pharmacokinetic analysis by orally administering resveratrol-βcd or resveratrol to mice at doses of 25 mg/kg (0.1 mmol/kg) or 50 mg/kg (0.2 mmol/kg). The results showed that at 50 mg/kg, the C_max_ value of the complex was 2.17 ± 0.27 ng/ml, while the control group had a C_max_ value of 0.24 ± 0.07 ng/ml, indicating a high peak concentration. There was also a significant difference in Area Under the Curve (AUC) between the two groups (resveratrol-βcd: 101,573 ± 2912 vs. resveratrol: 60,259 ± 1305, *p* = 0.0003) (Fig. 1D). The results of the 25 mg/kg group were consistent with those of the 50 mg/kg group (Fig. 1E). Furthermore, we evaluated the effects of resveratrol-βcd on the physiological and immune status of mice. We administered resveratrol-βcd at doses of 25 mg/kg or 50 mg/kg every other day via oral gavage. Resveratrol-βcd had a minimal effect on mouse body weight (Fig. 1F). Based on our previous research, resveratrol has a strong activating effect on dysfunctional macrophages. The activation state of macrophages was also evaluated during treatment. Over the observation period, the level of TNFα, an important cytokine derived from activated monocyte-macrophages, in peripheral blood showed minimal changes (Fig. 1G). On day 14 posttreatment, we euthanized the mice, and there were no significant differences in spleen size among the different groups (Fig. 1H, I).

### Resveratrol-βcd suppresses POI in a mouse model

**Fig. 2:**
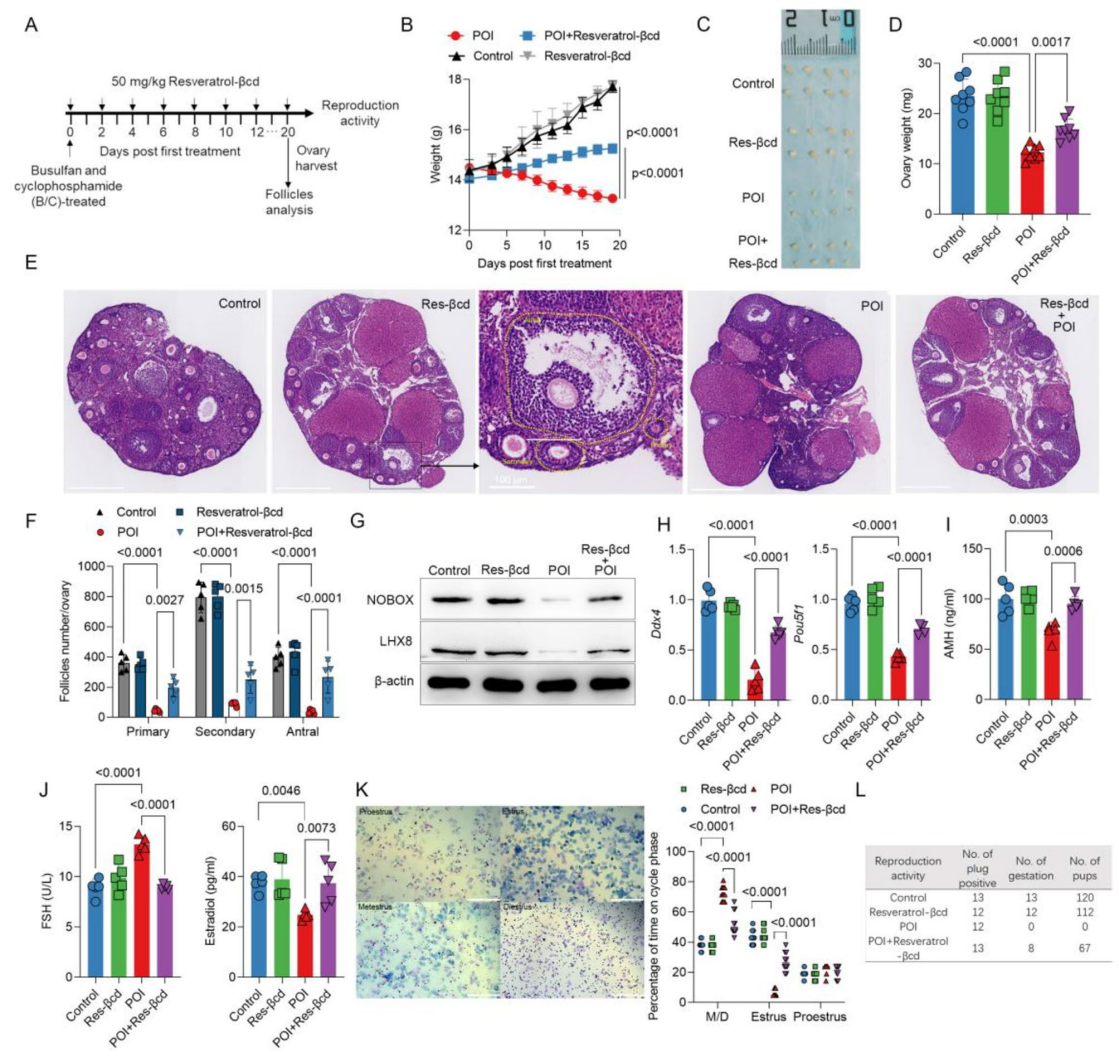
Resveratrol-βcd can inhibit the progression of POI in a mouse model. **(A)** Schematic diagram of resveratrol-βcd treatment; **(B)** Changes in body weight of the control group, negative control group (resveratrol-βcd alone), POI model group, and treatment group (POI + resveratrol-βcd) during the treatment, *n* = 5; **(C)** On the 20 day after treatment began, the ovaries of the mice were isolated and weighed **(D)**, with each point representing the weight of one ovary, *n* = 8; **(E)** Typical images of follicles at different stages in the ovaries, scale bar = 500 μm and the corresponding statistical analysis results **(F)**, *n* = 5; **(G)** Typical western blot result of LHX8 and NOBOX expression levels in ovarian tissue, *n* = 3, see the other two western blot replicates in Supplementary Fig. 2; All the repetitive results of western blot in this paper can be found in Supplementary Fig. 2, which will not be described afterwards; **(H)** Total RNA was extracted from ovarian tissue, and qPCR was used to analyze the transcriptional changes of *Ddx4* and *Pou5f1*, *n* = 5; **(I)** On day 20 post treatment, mouse serum was collected to detect AMH, FSH and E2 **(J)** levels in the blood, *n* = 5. **(K)** Estrous cycles examination by vaginal smears, scale bar = 200 μm, percentage of time on cycle phase was shown on the right, M/D = Metestrus and Diestrus, *n* = 10; **(L)** Reproductive activity analysis of different treatment group, *n* = 15; Statistical analysis was performed using two-way ANOVA **(B)**, and one-way ANOVA (**D,F,H,I,J** and **K**) comparison was made POI vs. control, POI vs. resveratrol-βcd and POI vs. POI + resveratrol-βcd; ns, not significant

Based on the resveratrol-βcd safety confirmed above, busulfan and cyclophosphamide (B/C)-treated mice, the POI mouse model, were treated with resveratrol-βcd at a dosage of 50 mg/kg every other day via oral gavage (Fig. 2A). The combination of cyclophosphamide and busulfan is frequently employed for creating a mouse model of Premature Ovarian Insufficiency (POI) due to its rapid model establishment and high success rate [19–22]. The results showed that the body weight of mice in the POI model group gradually decreased throughout the model construction period, while mice treated with resveratrol-βcd exhibited significant weight gain compared to the POI group (Fig. 2B). After the treatment period, the mice were dissected, and the weight of the ovaries was measured. Compared to the control group, the ovarian weight of mice decreased by 46.24% after B/C treatment, but there was significant recovery in ovarian weight after resveratrol-βcd treatment (Fig. 2C, D).

During the treatment process, we evaluated ovarian function-related phenotypes such as fertility rate, estrous cycle, and quantity of ovarian follicle, and also conducted tests on genes and proteins that showed a clear positive correlation with reproductive cell function. The use of resveratrol-βcd resulted in obvious recovery of the quantity of primary, secondary, and antral follicles in the ovaries (Fig. 2E, F). We also examined LIM homeobox gene 8 (LHX8) and newborn ovary homeobox (NOBOX), two proteins that are associated with ovarian development. LHX8 plays an important role in the formation and maintenance of primordial follicles as well as early follicle development [23]. Lack of NOBOX leads to accelerated loss of oocytes from primordial follicles to growing follicles and induces fibrosis in ovarian tissue in female mice [24]. The results showed that the expression levels of both proteins were moderately enhanced compared to those in the POI group after resveratrol-βcd treatment (Fig. 2G). Transcript levels of some typical markers in the ovary were also examined, and it was observed that the gene levels of *Ddx4* (also known as mouse vasa homolog) and *Pou5f1* (also known as octamer-binding transcription factor 4) exhibited significant recovery after treatment (Fig. 2H). The decrease in AMH in the blood was notably inhibited (Fig. 2I), and the level of FSH also significantly decreased (Fig. 2J), with a significant recovery seen in the level of E2 (Fig. 2J). After treatment, estrous cycles examination showed that the estrous cycle of POI mice underwent noticeable changes, with a significant reduction in the duration of the estrous phase, this condition was partially alleviated in the treatment group (Fig. 2K). After treatment, reproductive activity analysis was conducted on the treatment group and the POI group. 5 IU PMSG was injected intraperitoneally into the mice, and the vaginal plugs was examined the morning after mating. The POI mice with vaginal plugs did not produce fertilized embryos, indicating evident infertility symptoms in the POI mice. resveratrol-βcd treatment group showed partial restoration of this condition (Fig. 2L).

### Resveratrol-βcd maintained ovarian granulosa cell function and promoted GC autophagy

**Fig. 3:**
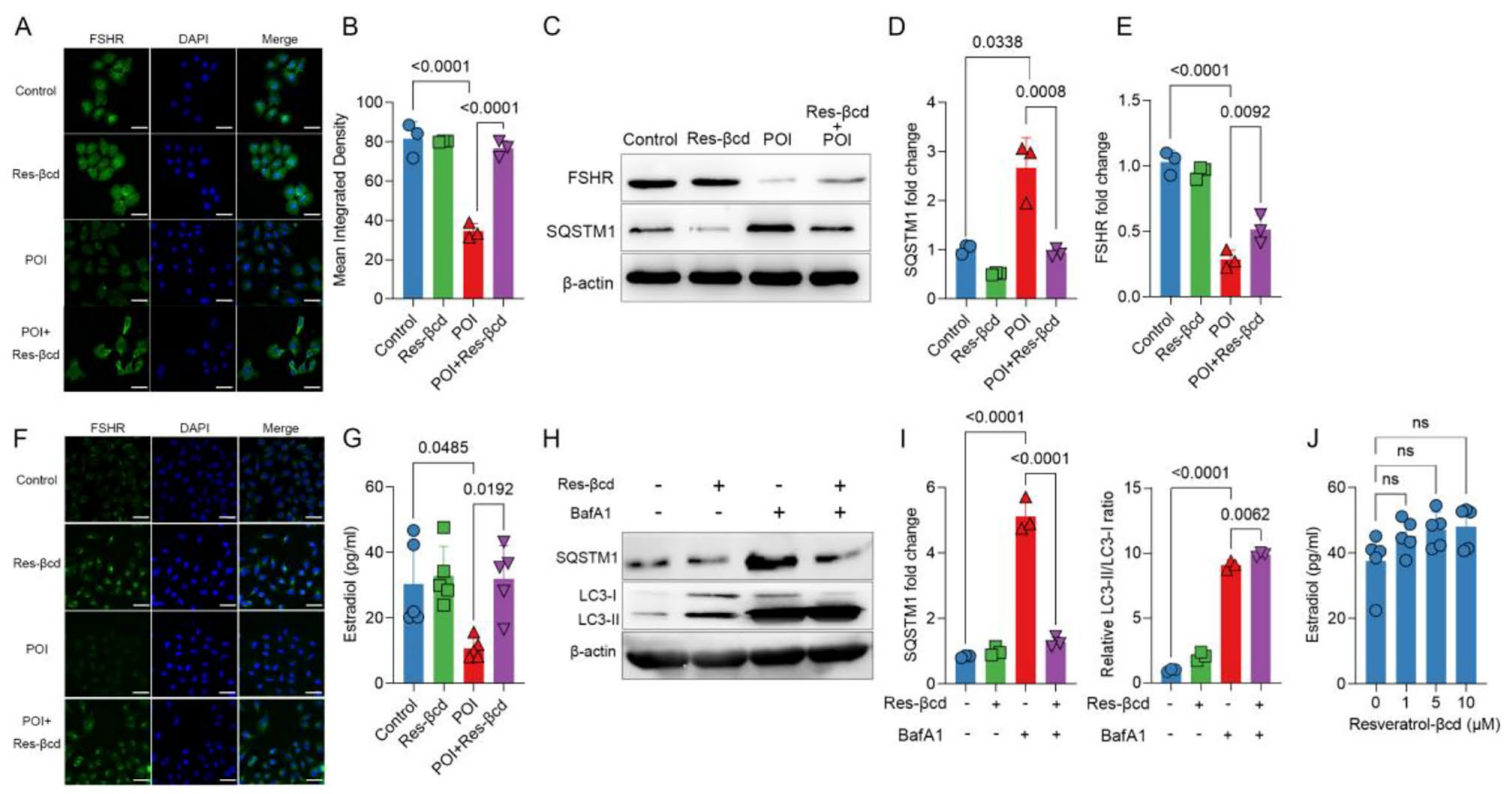
Resveratrol-βcd can maintain the function of granulosa cells during POI progression. **(A)** On day 14 post treatment, primary ovarian granulosa cells were isolated and stained for FSHR using the indirect immunofluorescence method, with green fluorescence representing FSHR and blue fluorescence representing cell nuclei. The imaging parameters were consistent for all fields of view, scale bar = 40 μm. The mean fluorescence intensity (MFI) values of green fluorescence in three random fields were statistically analyzed **(B)**; **C.** Typical western blot result and statistical analysis of SQSTM1 **(D)** and FSHR **(E)** expression levels in primary granulosa cells, *n* = 3; **(F)** The indirect immunofluorescence method was used to measure the level of LC3 in primary granulosa cells from different groups, scale bar = 40 μm; **(G)** A total of 1 × 10^5^ primary granulosa cells were cultured in a 12-well plate with phenol red-free DMEM/F12 medium (containing 10% charcoal-stripped fetal bovine serum), and 10 µM testosterone and 1 IU FSH were added. After 48 h of culture, the level of E2 in the supernatant was detected using the ELISA method, *n* = 5; **(H)** The lysosomal inhibitor bafilomycin A1 (BafA1) (100 nM) and 5 µM resveratrol-βcd were incubated alone or together with primary granulosa cells for 12 h, and western blotting was used to detect the expression levels of SQSTM1 and LC3. Statistical analysis is shown **(I)**, *n* = 3. **(J)** A total of 1 × 10^5^ primary granulosa cells was cultured in a 12-well plate and treated with the indicated concentrations of resveratrol-βcd. After 24 h of culture, 10 µM testosterone and 1 IU FSH were added. After 48 h of culture, the level of E2 in the supernatant was measured, *n* = 5. Statistical analysis was performed using one-way ANOVA (**B,D,E,G,I** and **J** comparison was made POI vs. control, POI vs. resveratrol-βcd and POI vs. POI + resveratrol-βcd; ns, not significant

E2 can be produced in various organs throughout the body, but it is mainly derived from ovarian granulosa cells and adipose tissue [25]. It has been proven that autophagy in GCs plays an important regulatory role in steroidogenesis [26]. Therefore, we analyzed the phenotypes and autophagy status of ovarian granulosa cells. Primary ovarian granulosa cells were isolated from the POI model or resveratrol-βcd treatment group. The levels of FSHR were measured using indirect immunofluorescence (Fig. 3A, B). The results showed a significant increase in FSHR levels in the treatment group. The FSHR western blot results were consistent with the indirect immunofluorescence results (Fig. 3C, E).

It has been reported that resveratrol can act as an autophagy activator and modulate cell metabolism and differentiation [27, 28]. We analyzed the autophagy levels of these cells, and the results showed that the autophagy levels derived from the POI mouse model were significantly inhibited (Fig. 3D, F), but there was a significant recovery in the autophagy status of primary ovarian granulosa cells after treatment with resveratrol-βcd. Additionally, GCs derived from the treatment group exhibited higher levels of E2 secretion (Fig. 3G). This suggests that resveratrol-βcd effectively maintains the normal function of GCs during the progression of POI.

Furthermore, we identified the activation of autophagy using resveratrol-βcd in vitro. After treatment with resveratrol, there was a certain elevation in the autophagy levels of primary granulosa cells. Western blot analysis showed an increase in the ratio of LC3II/LC3I and a decrease in SQSTM1/p62 levels (Fig. 3H, I). However, when primary granulosa cells were stimulated with different concentrations of resveratrol-βcd in vitro, the levels of E2 showed a slight increase, but there was no significant difference between groups (Fig. 3J), which is inconsistent with the results from in vivo experiments (Fig. 2L). This suggests that there may be other factors involved in the regulation of ovarian granulosa cells.

### Resveratrol-βcd can activate macrophages to secrete IL-6, antagonizing the process of POI

**Fig. 4:**
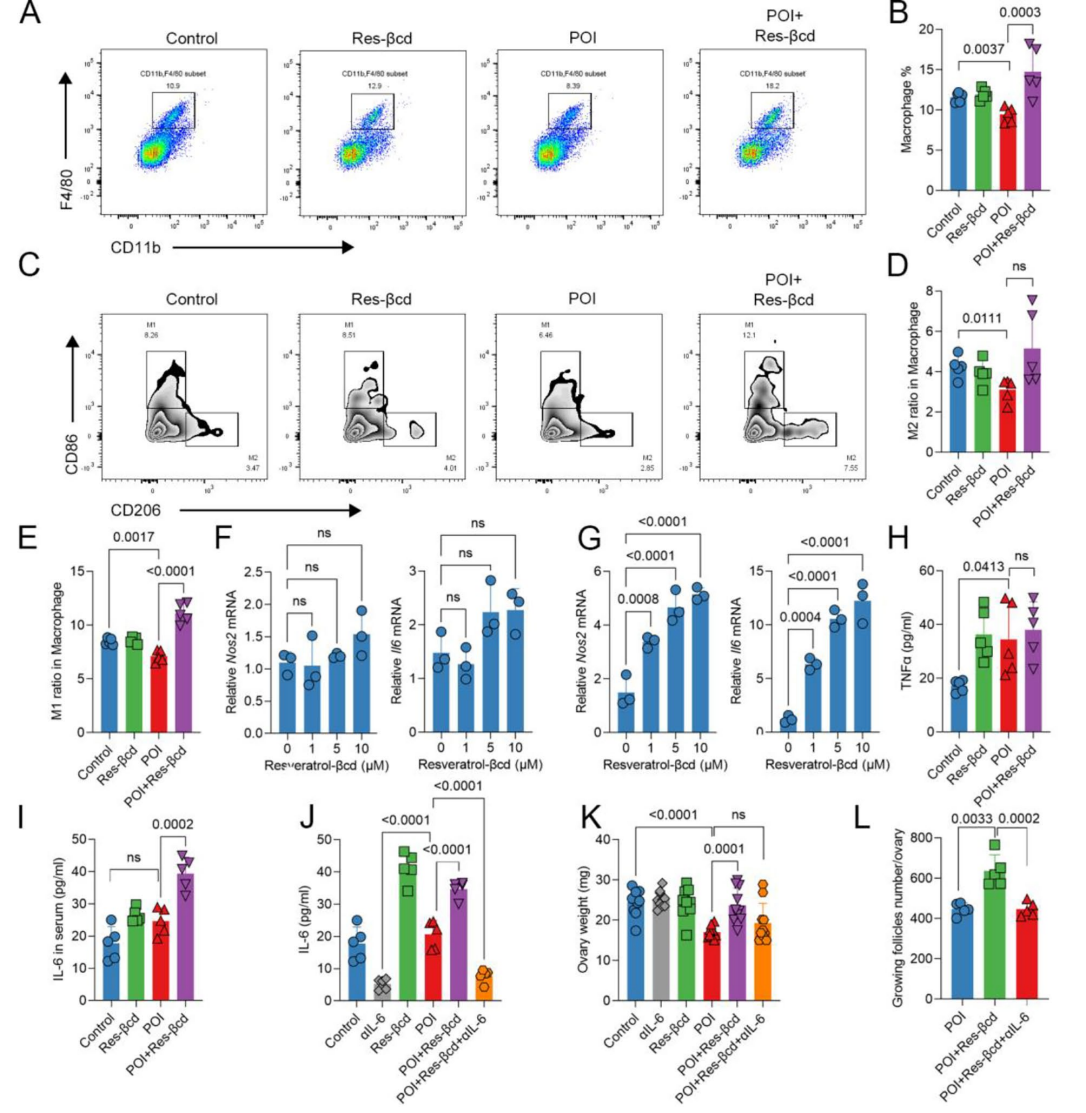
Resveratrol-βcd activates primary macrophages. **(A)** Representative flow cytometry dot plot of macrophages in ovarian tissue and the proportion of macrophages in different groups **(B)**, *n* = 5; **(C)** Representative flow cytometry contour plot of M1 and M2 macrophages differentiated by CD86 and CD206, as well as the proportion of M2-like **(D)** and M1-like **(E)** macrophages, *n* = 5; **(F)** RAW264.7 and primary macrophages from peritoneal sources **(G)** were cocultured with resveratrol-βcd at indicated concentrations for 24 h, and the transcription levels of *Nos2* and *Il6* were analyzed, *n* = 3; **(H)** On the 20th day of the treatment cycle, mouse serum was collected to measure the levels of TNFα and IL-6 **(I)** in peripheral blood, *n* = 5; **(J)** In the IL-6 neutralization experiment, mice were injected with 200 µg/each IL-6 antibody via tail vein at the same time point as the Resveratrol-βcd treatment, and on the day 20 post treatment began, the level of IL-6 in peripheral blood and ovary was detected, *n* = 5; **(K)** The ovaries of mice were dissected and weighed, with each point representing the weight of one side of the ovary, *n* = 10; **(L)** Statistical results of the number of all growing follicles in the ovaries, *n* = 5. Statistical analysis was performed using one-way ANOVA, comparison was made POI vs. each other groups in B,D,E,H,I,J,K and L; one-way ANOVA comparison was made 0 µM resveratrol-βcd vs. 1 µM, 5 µM or 10 µM resveratrol-βcd in F and G; ns, not significant

Our previous results showed that using resveratrol can modulate the relationship between macrophages and T cells [18]. We evaluated the changes in the proportion and polarization level of ovarian macrophages in the POI model and the resveratrol-βcd treatment group. The analysis of macrophage levels in the ovaries revealed a significant decrease in the macrophage ratio in the POI group. After treatment with resveratrol-βcd, there was a significant recovery in the macrophage ratio (Fig. 4A, B). Analysis of the M1 and M2 macrophage proportions showed a certain degree of decrease in the proportion of M2 macrophages in the POI mouse model. After treatment with resveratrol-βcd, the proportion of M2 macrophages exhibited some recovery, but there was no statistical significance (Fig. 4C, D). The proportion of M1 macrophages significantly increased (Fig. 4E). We evaluated the macrophage activation ability of resveratrol-βcd in vitro. The activating effect of resveratrol-βcd on RAW 264.7 cells, a macrophage-like cell line, was minor (Fig. 4F), but resveratrol-βcd directly promoted the differentiation of primary macrophages into the M1 phenotype (Fig. 4G). Apart from transcription markers, we analyzed the cytokines that may be derived from macrophages in serum and ovarian cells during the treatment process of the POI mouse model. The level of TNFα in the serum of POI mice showed a certain increase. In the resveratrol-βcd treatment group, there was no significant difference in TNFα levels compared to those in the POI group (Fig. 4H). Interestingly, after treatment with resveratrol-βcd, the level of IL-6 showed a significant increase (Fig. 4I).

To elucidate the role of IL-6 in antagonizing the process of POI during resveratrol-βcd treatment, we conducted neutralization experiments using an anti-IL-6 antibody (αIL-6). The results showed that the anti-IL-6 antibody could significantly reduce IL-6 levels in peripheral blood (Fig. 4J). αIL-6 partially neutralized the effects of resveratrol-βcd. On day 20 posttreatment, the ovarian weight and the number of growing follicles in the αIL-6 neutralization group were significantly lower than those in the group treated with resveratrol-βcd alone (Fig. 4K, L).

### Resveratrol-βcd and IL-6 can synergistically promote autophagy

**Fig. 5:**
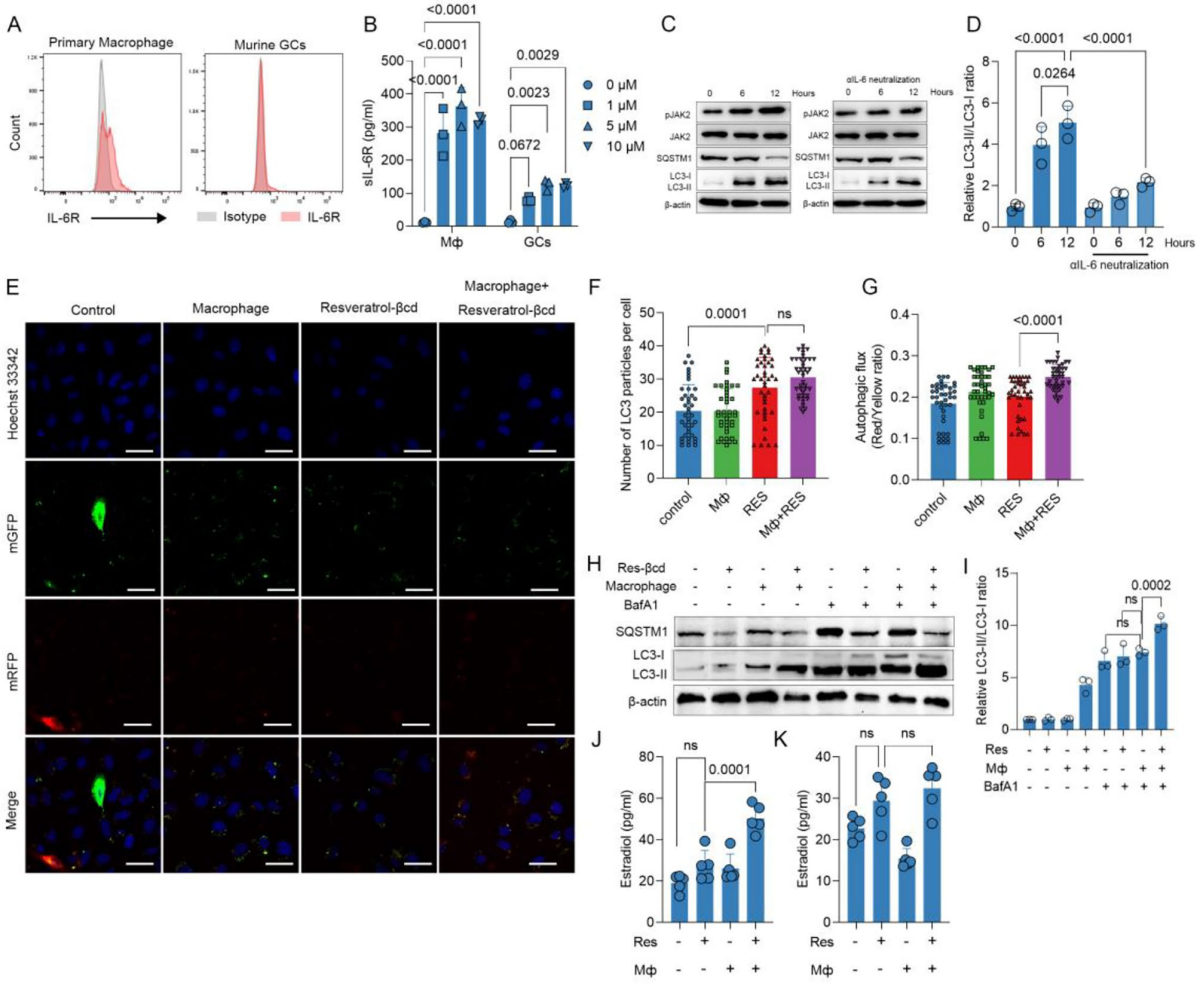
Resveratrol-βcd synergistically promotes autophagy in ovarian granulosa cells via the IL-6/sIL-6R pathway. **(A)** IL-6R levels on the surface of ovarian granulosa cells and primary macrophages; **(B)** After stimulating primary macrophages and ovarian granulosa cells with different concentrations of resveratrol-βcd for 24 h, culture medium was collected, and the levels of sIL-6R in the supernatant were detected using ELISA, *n* = 3; **(C)** Typical western blot result of the activation of JAK2 and protein levels of autophagy markers SQSTM1 and LC3 under different culture conditions, LC3-II/LC3-I and statistical analysis is shown in **(D)**, *n* = 3; **E.** Typical images of primary granulosa cells transfected with mRFP-mGFP-LC3 lentivirus and cultured with different treatments for 24 h; **(F)** Quantification of the number of mRFP-mGFP-LC3 puncta from **(E)** and the ratio of autolysosomes (red, mRFP + GFP-) to autophagosomes (yellow, mRFP + GFP+) **(G)**, *n* = 40 for each group. **(H)** Typical western blot results of SQSTM1 and LC3 under different culture conditions. LC3-II/LC3-I statistical analysis is shown in **(I)**, *n* = 3. **(J)** After coculturing with macrophages and/or resveratrol or with anti-IL-6 antibody **(K)**, the level of E2 in the supernatant of primary granulosa cells was measured after 48 h, *n* = 5. Statistical analysis was performed using one-way ANOVA, comparison was made 0 µM resveratrol-βcd vs. 1 µM, 5 µM or 10 µM resveratrol-βcd in B; comparison was made 12 h post IL-6 mono-treatment vs. each other groups in D; comparison was made resveratrol-βcd mono-treatment group vs. each other groups in **F, G, J, K**; comparison was made macrophage + BafA1 group vs. each other groups in **I**; ns, not significant

IL-6 is a pleiotropic cytokine that participates in the physiological activities of almost all organ systems. Recent studies have shown that IL-6 can induce autophagy [29]. IL-6 belongs to the IL-6 cytokine family, and its pathway is mediated through glycoprotein 130 (gp130) [30]. IL-6 cannot directly activate gp130 and must first bind to IL-6R, a component of a pathway known as the classic pathway [31], to further activate intracellular signaling, such as the JAK/STAT pathway [30]. However, only a few cell types, including liver cells and several subsets of leukocytes, express IL-6R on their cell surface. To clarify whether IL-6 can directly act on ovarian granulosa cells. We first analyzed the IL-6R level on the surface of primary ovarian granulosa cells, which was found to be relatively low (Fig. 5A). In addition to cell surface IL-6R, there is also a soluble form of IL-6R (sIL-6R), which has similar affinity to IL-6 compared to membrane-bound IL-6R, and the IL-6/sIL-6R complex can induce the formation of gp130 homodimers on almost all cell types, a process known as IL-6 trans-signaling [32]. Our results showed that consistent with previous reports [33], resveratrol-βcd can induce the secretion of sIL-6R from primary macrophages (Fig. 5B). At the same time, resveratrol-βcd weakly stimulated the secretion of sIL-6R by primary granulosa cells in vitro (Fig. 5B). To further determine whether the IL-6/sIL-6R signaling pathway is involved in the effects of resveratrol-βcd on GCs, we also identified downstream signaling pathways. Resveratrol-βcd enhanced the phosphorylation level of JAK2 over time in the coculture of primary granulosa cells and macrophages in vitro (Fig. 5C). When neutralizing with IL-6 antibody, the coculture of primary granulosa cells and macrophages failed to activate the JAK2 signaling pathway in primary granulosa cells (Fig. 5C). We also observed that during coculture, the autophagy level of granulosa cells was increased with the activation of JAK2 (Fig. 5C, D). We further evaluated the impact of resveratrol-βcd on autophagic flux in GCs by using mRFP-GFP-LC3 lentivirus-transfected primary granulosa cells. There was a significant increase in the number of LC3 puncta in both the resveratrol-βcd alone group and the coculture groups with resveratrol-βcd and macrophages (Fig. 5E, F). The ratio of red to yellow puncta was significantly enhanced after adding macrophages, indicating a further synergistic effect of autophagic flux by macrophages and resveratrol-βcd (Fig. 5G).

To confirm the synergistic effect of resveratrol-βcd and IL-6 in promoting autophagy, we stimulated granulosa cells derived from (B/C)-treated mice with IL-6 and resveratrol-βcd together, and the results showed that macrophage-derived cytokines and resveratrol-βcd further enhanced the level of autophagy (Fig. 5H, I). When macrophages were added, the secretion of E2 by primary granulosa cells was significantly enhanced (Fig. 5J), and this promoting effect was inhibited when IL-6 antibody was added (Fig. 5K). In conclusion, macrophage-derived IL-6 stimulated by resveratrol-βcd can synergistically enhance the autophagic function of granulosa cells and E2 secretion.

## Discussion

The function of resveratrol in promoting autophagy has been validated in various types of cells. In vitro experiments have shown that resveratrol can directly inhibit mammalian target of rapamycin (mTOR) [34], a key regulator of autophagy [35]. Resveratrol can enhance the interaction between mTOR and the mTOR inhibitor DEPTOR (DEP-domain containing mtor-interaction protein) [36, 37, 29, 39]. Resvega, a commercial product containing resveratrol, has been shown to upregulate autophagic flux and autolysosome formation [27]. These results suggest that resveratrol could promote the autophagic pathway in GCs. In this study, our results demonstrate that resveratrol can directly act on primary granulosa cells and promote their autophagy.

On the other hand, resveratrol can also exert its antagonistic effect on POI by modulating the immune response [12]. In infectious disease research, resveratrol has been shown to activate M2 macrophages during pathogen infection [38–40]. Our previous report also showed that resveratrol can balance the functions of macrophages and T cells [18]. We improved the stability of resveratrol by constructing resveratrol-βcd, making it easier for the body to absorb. In this experiment, the use of beta-cyclodextrin increased the C_max_ value of resveratrol in vivo (Fig. 1D, E), which is beneficial for enhancing its local effects on the body.

Many groups have extensively studied the ovarian immune microenvironment in patients with primary ovarian insufficiency. In the peripheral blood of POI patients, there is a significant decrease in the proportion and proliferation level of Tregs [41]. However, the levels of IL-10, IL-4, and TGF-β in the serum of POI patients do not significantly differ from those of healthy controls [41]. This suggests that immune dysfunction caused by POI may only exist in the ovary. In the mouse model of POI, there is a high Th1 response level in the ovaries, and the high expression of IFN-gamma and TNF-alpha leads to granulosa cell apoptosis. In this study, the peripheral blood levels of TNF-alpha in POI mice did not show a significant increase (Fig. 4H), but we observed a significant increase in IL-6 levels in both peripheral blood and ovaries after resveratrol treatment (Fig. 4I, J).

 [44–50]IL-6 is a multifunctional cytokine, and the role of IL-6 in human oocyte maturation and subsequent embryo development is still uncertain. Some studies have suggested that high levels of IL-6 in follicular fluid are beneficial for oocyte maturation [42, 52]. However, other studies have drawn opposite conclusions: higher levels of IL-6 during in vitro fertilization cycles are associated with poorer embryo quality and lower chances of pregnancy [43, 44]. There are also studies suggesting that IL-6 does not affect the clinical pregnancy rate in IVF-ET and therefore does not affect oocyte quality [45, 46]. Our findings indicate that IL-6 exerts its effects on ovarian granulosa cells via the IL-6/sIL-6R pathway, as there is a lack of IL-6R on GCs (Fig. 5A), while sIL-6R derived from macrophages can act on GCs (Fig. 5B-C). It has been reported that *IL6Rα* mRNA and protein are highly expressed in granulosa cells of progressing preovulatory follicles [47, 48]. Therefore, the role of IL-6 may be discussed separately at different stages of follicle development. Detection of the dynamic changes in IL-6/IL-6R and their function during follicle development requires further investigation in subsequent studies.

There have been reports suggesting that IL-6 inhibits FSH-induced production of estradiol and progesterone in granulosa cells [49]. However, our results demonstrate that IL-6 generated after resveratrol-βcd treatment can enhance autophagy levels in granulosa cells and promote their activation (Fig. 5C). Consistently, the use of IL-6 antibodies inhibited the ability of resveratrol-βcd to antagonize POI (Fig. 4K, L). The inconsistent results compared to previous reports may be due to phenotypic differences in granulosa cells at different stages of follicular development or differences in mouse strain.

 [43]It is worth noting that the cell-specific effect of interleukin production is another important function of resveratrol. Resveratrol can increase the expression of IL-1β and IL-6 in peripheral blood lymphocytes [50], but its stimulating effect on cell lines such as RAW 264.7 is not significant [50]. This is consistent with the results of our study (Fig. 4F, G), where resveratrol-βcd failed to significantly activate RAW 264.7 cells in vitro. The enhancement of IL-1β and IL-6 production is characteristic of proinflammatory states, which aids in the differentiation and function of T helper lymphocytes [51], but it also plays a role in tissue regeneration [52]. These results suggest that resveratrol-βcd may have specific receptors or signaling pathways to regulate cell development, and further research on resveratrol could focus on the signaling pathways and resveratrol-Treg regulation.

Our research also has certain limitations. IL-6, as a multifunctional cytokine [53], not only induces autophagy but also has profound effects on other cells within the ovary and ovary functions [54]. Additionally, the function of resveratrol is not solely restricted to stimulating macrophages; it can directly impact some cellular signaling pathways [10, 11]. In this study, we were unable to provide sufficient clarification regarding the interrelationships among these potential mechanisms. This will be a target for subsequent research.

## Methods

### Construction and stability identification of resveratrol-βcd

Resveratrol-βcd was prepared according to previous literature [18]. Briefly, at room temperature, resveratrol (MCE, HY-16,561) was added to a sulfobutyl ether-β-cyclodextrin solution (βcd, Shanghai Chineway Pharmaceutical Tech. Co., Ltd.) in excess of its intrinsic solubility. The mixture was sonicated and stirred in a water bath. After filtration through a 0.22 μm filter, the solution was freeze-dried. The stoichiometry of the complex was determined by Job’s plot method, showing a maximum value at *R* = 0.5 and a symmetrical shape, indicating a 1:1 complex. Resveratrol-βcd was dissolved in PBS, with a storage concentration of 3 mg/ml, PBS was used for dilution for subsequent in vivo experiments. The resveratrol concentration in the culture medium or blood was measured using a Resveratrol ELISA Kit (Cloud-Clone Corp., Wuhan, China) following the manufacturer’s instructions. For mouse serum resveratrol measurement, mouse blood was collected into a 1.5 ml tube, allowed to stand at 37 °C for 60 min, and then stored at 4 °C overnight. Centrifugation at 500 × g for 5 min at 4 °C was performed to collect the upper layer serum for resveratrol-βcd detection.

### Cell lines and animals

3-week-old (10–12 g) or 8-week-old (20–22 g) (depending on the experimental requirements) female C57BL/6 mice were obtained from Shanghai Model Organisms Center, Inc. For 8-week-old mice, we confirmed the normal estrous cycle by vaginal smears and housed them in a specific pathogen-free (SPF) animal facility with controlled environmental conditions (22–24 °C, 60–70% relative humidity, 12-hour light-dark cycle) and free access to food and water. These animals were cared for following the “Principles of Laboratory Animal Care” guidelines. All experimental procedures involving animals were approved by the Ethics Committee of Shenzhen TopBiotech Co., Ltd.

The COV434 cell line and RAW 264.7 cells were obtained from Procell Life Science & Technology Co., Ltd. (Wuhan, China) and cultured in DMEM supplemented with 10% FBS, 2 mM glutamine and 1% P/S (100 mg/ml streptomycin sulfate and 100 U/ml penicillin). The cells were maintained in a saturated atmosphere of 37 °C, 95% air, and 5% CO2 and tested negative for mycoplasma contamination. The RAW264.7 cells used in this experiment were passages 6 to 8, and the COV434 cells were passages 8 to 10.

### POI mouse model construction

The POI mouse model was established by a single intraperitoneal injection of busulfan (30 mg/kg) (Sigma, B2635) and cyclophosphamide (120 mg/kg) (MCE, HY-17,420). The day of administration was recorded as day 1 after the first treatment. Resveratrol-βcd was orally administered at a dose of 50 mg/kg every other day. Each group used five 8-week-old mice for experiment, prior to resveratrol-βcd administration, each mouse was weighed. In the IL-6 neutralization experiment, 200 µg/mouse IL-6 antibody (Bioxcell, BE0046) was administered via intravenous injection at the same time points as resveratrol-βcd. Reproductive activity analysis was performed in the 8th week after treatment, 5 IU PMSG (Solarbio, P9970) was injected intraperitoneally into the mice, and the vaginal plugs was examined the morning after mating. Each group used 5 mice for experiment, in vivo experiments were independently repeated three times.

### Primary mouse granulosa cell isolation

The isolation of primary granulosa cells was performed as previously reported [55, 56] with slight modifications. 3-week-old female C57BL/6 mice were euthanized and sterilely dissected to remove the ovaries. The excised ovaries were placed in DMEM/F12 medium containing 1 mg/ml bovine serum albumin and 1% P/S. The tissues surrounding the ovaries were cleaned under a dissecting microscope (Leica, Singapore, M125) and washed twice using the aforementioned medium. GCs were collected by puncturing the excised ovaries with a 25-gauge needle. Cells were resuspended in DMEM/F12 medium (without phenol red) supplemented with 10% charcoal-stripped fetal bovine serum (Vivacell, C3830) and 1% P/S. Cell viability was determined by trypan blue staining, and the purity of granulosa cells was identified using an FSHR antibody (Proteintech, 22665-1-AP). Cells were cultured in basal medium (without phenol red) alone or with different concentrations of testosterone (Sigma, T1500) and FSH for 48 h at 37 °C in a saturated water vapor atmosphere with 95% air and 5% CO_2_. The culture medium was collected for E2 measurement through an electrochemical method.

### Primary macrophage isolation and transwell coculture

The isolation of primary macrophages was performed as previously described [57]. Briefly, mice were euthanized using cervical dislocation and sterilized with 70% ethanol. The abdominal wall was opened to expose the peritoneum and sterilized again with 70% ethanol. Using sterile forceps, 10 ml of sterile PBS was injected into the posterior side of the abdomen, gently shaking the whole body for 10 s and slowly withdrawing the saline containing peritoneal cells. The cells were resuspended in macrophage culture medium (RPMI 1640 with 10% FBS, 50 IU penicillin, 50 µg streptomycin, and 2 mM glutamine) and incubated at 37 °C for 60 min. After incubation, the cells were washed five times with preheated PBS to remove nonadherent cells.

### Cell proliferation was assessed using the CCK8 assay

**A** 1 × 10^4^ cell/100 µL/well cell suspension was prepared in a 96-well plate. The plate was incubated in a CO_2_ incubator for 24 h at 37 °C. Then, 5 µM resveratrol-βcd or resveratrol was added to the plate, and the medium with the drug was replaced every 24 h for 4 days. On days 1, 2, 3, and 4 after drug treatment, the Cell Counting Kit-8 (CCK-8) assay (YEASEN, China) was used to measure cell proliferation. Before adding the CCK-8 solution, the culture medium was removed, and the cells were washed twice with medium to remove any residual drug. Then, 10 µL of CCK-8 solution was added to each well, and the plate was incubated in a CO_2_ incubator for 2 h. The absorbance at 450 nm was measured.

### Enzyme-linked immunosorbent assay (ELISA)

For the detection of cytokines and steroid hormones, corresponding ELISA kits were used according to the manufacturer’s instructions. For mouse serum samples, whole blood without anticoagulant was collected and incubated at 37 °C for 30 min, followed by centrifugation at 500 × g for 5 min to obtain the supernatant. The serum was diluted 1:1 with PBS before use as the sample for ELISA. Three replicate wells were set up for each sample. The following ELISA kits were used: Mouse IL-6 (Biolegend, 431,304), Mouse TNF-α (Biolegend, 430,904), Mouse FSH (Follicle Stimulating Hormone) ELISA Kit (Elabscience, E-EL-M0511C), Mouse AMH (Anti-Mullerian Hormone) ELISA Kit (Elabscience, E-EL-M3015), and sIL-6 Mouse IL-6R alpha duoset ELISA (R&D, DY1830). The absorbance at 450 nm was measured.

### Flow cytometry

The single-cell suspension was prepared using mechanical dissociation. Briefly, the isolated ovaries were thoroughly washed with PBS, and the fatty tissue was removed. The ovaries were then placed on a 70 μm cell filter (Biosharp, BS-70-XBS), and the tissue was ground with the rubber head of a 5 ml syringe to obtain a single-cell suspension. The prepared single-cell suspension was washed once with PBS and stained with the following monoclonal antibodies for cell surface phenotypic analysis: CD45-efluor 450 (eBioscience, 48-0451-82), CD11b-APC cy7 (eBioscience, 47-0112-82), F4/80-PE (eBioscience, 12-4801-82), CD86-FITC (eBioscience, 11-0862-82); CD206-APC (eBioscience, 17-2061-82), and IL-6R-PE (eBioscience, 12-1261-80). For IL-6R, rat IgG2b kappa isotype control (eB149/10H5) and PE (eBioscience, 12-4031-82) were used as isotype controls. The single-cell suspension was stained with antibodies for 30 min at 4 °C in the dark, and single-color controls were also prepared for compensation adjustment. The samples were washed and resuspended in PBS. Flow cytometry data acquisition was performed using Cytoflex LX (Beckman Coulter, Brea, CA). Data analysis was conducted using FlowJo 10.0. Supplementary Fig. 3 provides an example of the gating strategy.

### Histological section preparation and HE staining

After the treatment was finished, the mice were euthanized, and the ovaries were isolated. The ovaries were fixed in 4% paraformaldehyde at 4 °C for 24 h, with one change of fixative at 12 h after fixation. The ratio of fixative to tissue was 20:1. The ovaries were dehydrated using a gradient of 20% sucrose/PBS and 30% sucrose/PBS, with each gradient lasting 24 h and one solution change in between. The ovaries were embedded in O.C.T. Compound (Sakura, 4583), and the ovarian tissues were sectioned completely at a thickness of 5 μm. After air-drying at room temperature, the sections were washed with PBS for 5 min, stained with hematoxylin-eosin (HE) for 5 min, differentiated in 1% hydrochloric acid alcohol, rinsed in water for 30 min, counterstained with eosin for 5 min, dehydrated with 95% ethanol, absolute ethanol, and xylene (absolute ethanol: xylene = 1:1), and then mounted and observed using an upright microscope (Leica DM4B, Germany). The number of follicles in the ovaries was counted.

### Quantitative real-time PCR (qPCR)

The ovarian tissues were thoroughly minced, and total RNA was extracted using the AG steadypure Universal RNA Extraction Kit (AG, AG21017). The RNA concentration was measured using a NanoDrop 2000. Reverse transcription was performed using the Hifair III 1st Strand cDNA Synthesis supermix for PCR (Yeasen 11137ES10) according to the instructions. qPCR was conducted using Hieff qPCR SYBR Green Master Mix (No Rox) (YEASEN 11201ES08) on the Bio-Rad CFX96 System. The results were analyzed using the ΔΔCT method [58]. The primers used were as follows:

*Ddx4*: F: GGACGAGATTTGATGGCTTGTGC; R: AGCGACTGGCAGTTATTCCATCC;

*Pou5f1*: F: CAGCAGATCACTCACATCGCCA; R: GCCTCATACTCTTCTCGTTGGG;

*Il6*: F: TACCACTTCACAAGTCGGAGGC; R: CTGCAAGTGCATCATCGTTGTTC;

*Nos2*: F: GAGACAGGGAAGTCTGAAGCAC; R: CCAGCAGTAGTTGCTCCTCTTC;

*Gapdh*: F: CATCACTGCCACCCAGAAGACTG; R: ATGCCAGTGAGCTTCCCGTTCAG;

### Indirect immunofluorescence

Isolated primary ovarian granulosa cells were plated on cell slides and incubated overnight at 37 °C with 95% air and 5% CO2. The culture medium was removed, and the cells were washed once with precooled PBS. Cells were fixed with 4% paraformaldehyde at room temperature for 15 min. Then, the cells were blocked with 5% BSA/0.3% Triton X-100/PBS for 60 min. Next, FSHR antibody (Proteintech, 22665-1-AP) was added at a 1:50 dilution and incubated overnight at 4 °C. After washing three times with PBS, the cells were incubated with a secondary antibody (anti-rabbit Alexa Fluor 488, A32731, Invitrogen) diluted in antibody dilution buffer (1% BSA/0.3% Triton X-100/PBS) at a ratio of 1:2000 for 60 min at room temperature in the dark. Following three washes with PBS, cell slides were mounted with mounting medium with DAPI - Aqueous, Fluoroshield (Abcam, ab104139) and observed using a confocal microscope (ZEISS, LSM 880).

### Western blot

Cultured cells were washed once with precooled PBS. Then, 100 µl of RIPA buffer (Beyotime, PC101) (prior to use, PMSF was added to a final concentration of 1 mM) was added to each well of a 6-well plate and incubated on ice for 1 min. All the liquid was transferred to a new tube. The protein concentration of the lysate was determined using a BCA Protein Concentration Assay Kit (Beyotime, P0012). Then, 5X loading buffer (250 mM Tris-Cl pH = 6.8, 10% SDS, 0.5% bromophenol blue, 50% glycerol, 5% β-mercaptoethanol) was added to the lysate, and the mixture was incubated at 95 °C in a water bath for 5 min to prepare the samples for SDS‒PAGE.

A 12% or 15% concentration separating gel was used for SDS‒PAGE. The proteins were transferred to PVDF membranes using a semidry transfer system (Bio-Rad Trans-Blot Turbo). The membrane was blocked with 5% BSA/TBST at room temperature for 1 h. Dilute primary antibodies in 5% BSA/TBST and incubate overnight at 4 °C with shaking. The primary antibodies used in this experiment included LHX8 (Sigma, SAB2101342), NOBOX (Sigma, SAB2105362), SQSTM1 (CST, #88,588), LC3I/II (LC3A/B CST, #12,741), β-actin (Proteintech, 20536-1-AP), JAK2 (CST, #3230), and p-JAK2 (CST, #3771). The membrane was washed four times with 15 ml TBST for 5 min each time. Secondary antibodies were diluted in 5% BSA/TBST and incubated at room temperature for 60 min. The secondary antibodies used in this study were HRP-linked anti-rabbit IgG (CST, #7074) and HRP-linked anti-mouse IgG (CST, #7076). The membrane was washed four times with 15 ml TBST for 5 min each time. Following the instructions of Supersignal West Pico Plus Chemiluminescent Substrate (Thermo Scientific, 34,580), Solution A and Solution B were mixed at a 1:1 ratio, and the membrane was immersed in the mixture, incubated for 3 min, and visualized using the iBright FL1000 Imaging System (Thermo Fisher Scientific Inc.).

### Statistical analysis

All in vitro and in vivo experiments were independently repeated at least twice, and the data were analyzed by GraphPad 9.0.0 software (GraphPad). Statistical analysis was performed using unpaired two-tailed Student’s t test for two-group comparisons, two-way ANOVA was used for more than two-group comparisons, and *P* values < 0.05 were considered statistically significant, ns = not significant, Error bar = ± S.D.

### Electronic supplementary material

Below is the link to the electronic supplementary material.

Supplementary Material 1

## Data Availability

The datasets used and/or analysed during the current study are available from the corresponding author on reasonable request.
