# Supplementary material for: Resveratrol-βcd inhibited premature ovarian insufficiency progression by regulating granulosa cell autophagy
Source: J Ovarian Res. 2024 Jan 15;17:18. doi: 10.1186/s13048-024-01344-0 (PMC10789063; doi:10.1186/s13048-024-01344-0)
Supplement: Supplementary file 1 — Supplementary Material 1 [file 13048_2024_1344_MOESM1_ESM.docx]

Supplementary Material

**Supplementary Fig 1.** Resveratrol-βcd measurement using the resveratrol ELISA kit. The results showed that the resveratrol ELISA kit provided accurate measurements of resveratrol-βcd concentrations, with 200 ng/ml, 500 ng/ml, 2000 ng/ml, 4000 ng/ml, and 8000 ng/ml of resveratrol-βcd or resveratrol used for establishing the standard curve, n=5.


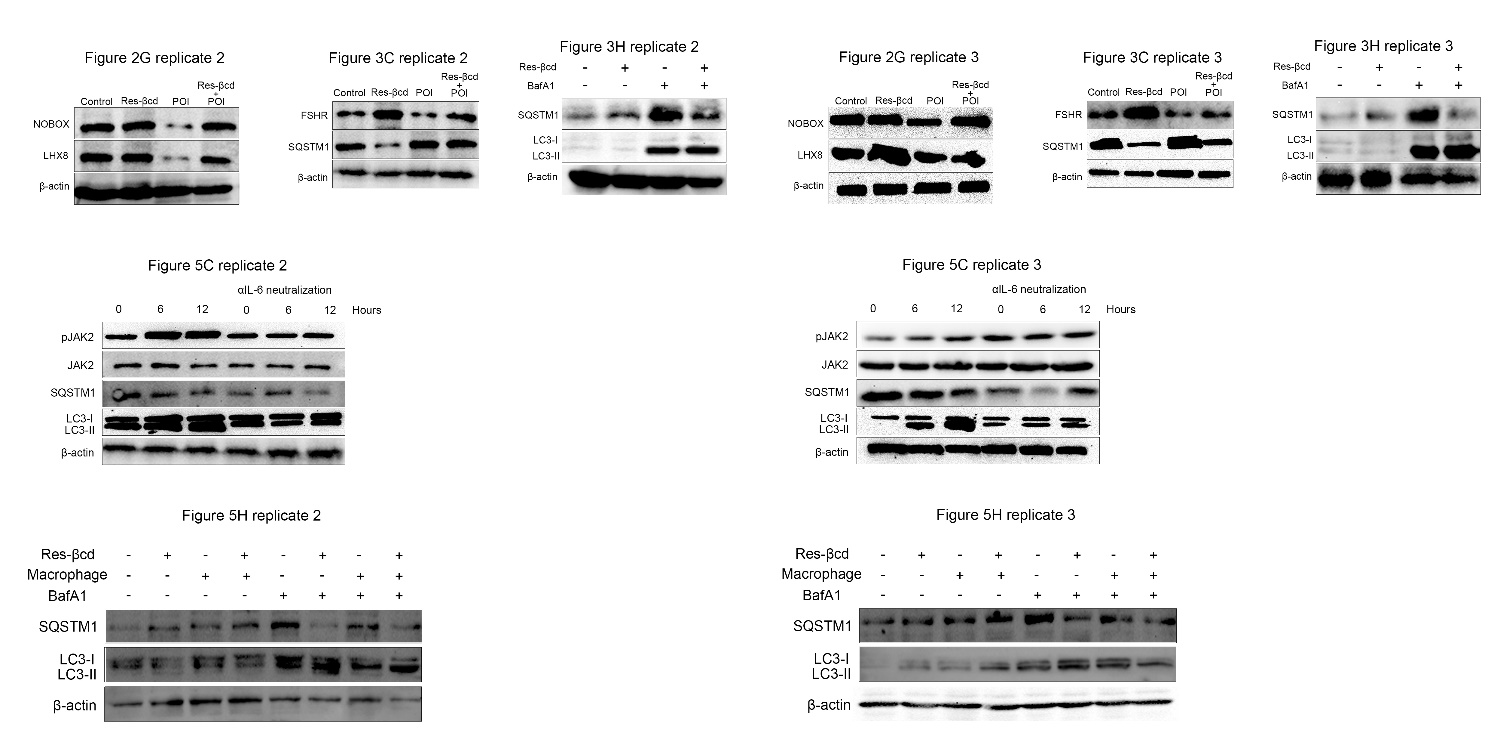


**Supplementary Fig 2.** Replication data of western blot shown in main figure.


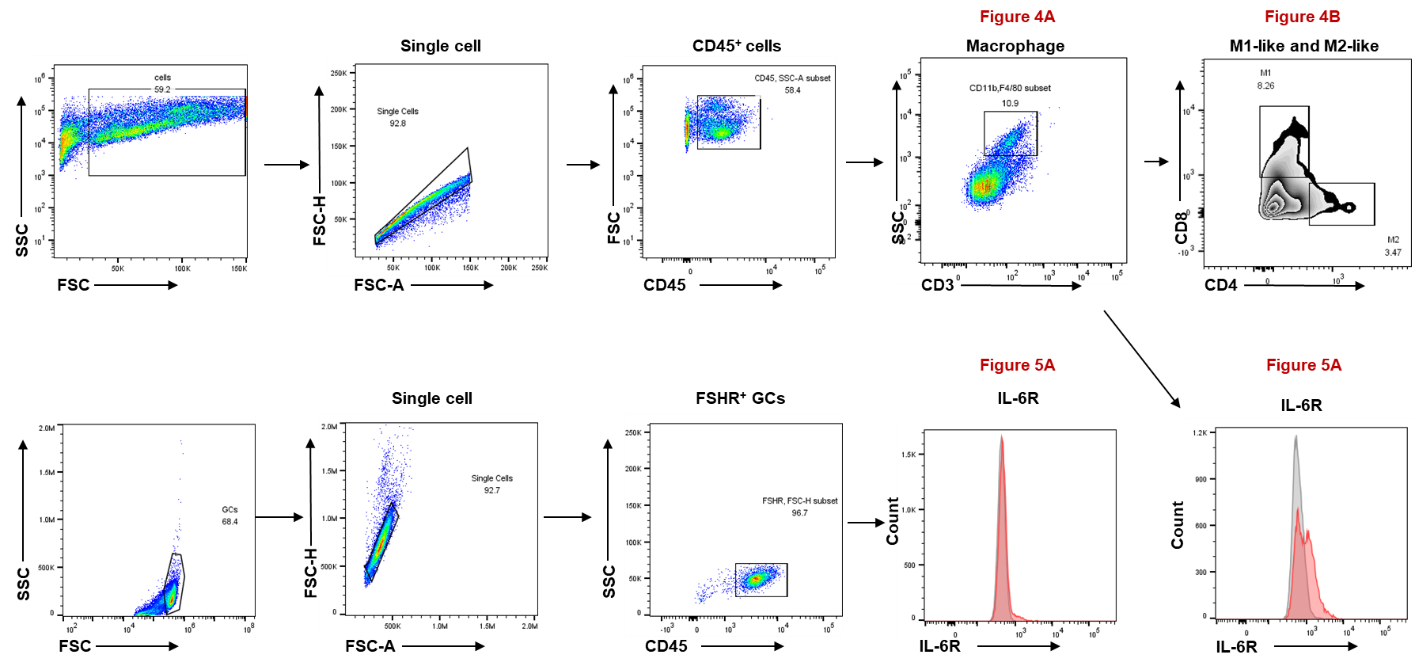


**Supplementary Fig. 3** Exemplifying gating strategies for FACS analysis are shown. Gating strategy for macrophage in ovary (for Figure 4A, 4B); gating strategy for isolated GCs (for Figure 5A).
